# *M. tuberculosis* Secretory Protein ESAT-6 Induces Metabolic Flux Perturbations to Drive Foamy Macrophage Differentiation

**DOI:** 10.1038/srep12906

**Published:** 2015-08-07

**Authors:** Varshneya Singh, Charanpreet Kaur, Vijay K. Chaudhary, Kanury V. S Rao, Samrat Chatterjee

**Affiliations:** 1 Drug Discovery Research Centre, Translational Health Science & Technology Institute NCR Biotech Science Cluster, 3rd Milestone, Faridabad - Gurgaon Expressway, Faridabad - 121001, India; 2 Department of Biochemistry, University of Delhi South Campus, New Delhi, 110021, India; 3 Immunology Group, International Centre for Genetic Engineering and Biotechnology, New Delhi, 110067, India

## Abstract

The Foamy Macrophage (FM) differentiation forms a major component of the host dependent survival axis of *M. tuberculosis*. The FM which are characterized by the intracellular accumulation of lipid bodies (LBs), ensure a privileged existence for the bacilli through ready provision of nutrients and by conferring protection against bactericidal pathways. The mycobacterial secretory protein ESAT-6 has been identified as the molecular mediator of the FM differentiation process although little is known about the mechanism through which it induces this process. In the present study, we show that ESAT-6 induces GLUT-1 mediated enhanced glucose uptake by macrophages which is coupled to metabolic flux perturbations in the glycolytic pathway caused by differential rates of reaction at several steps in the pathway. Two major changes identified were the simultaneous buildup of DHAP (for Triglyceride synthesis) and AcCoA (for synthesis of 3-HB, ligand for the anti-lipolytic GPR109A). We also show that part of the observed effects involve protein- protein interactions between ESAT-6 and the macrophage glycolytic enzymes, Enolase1 and Phosphoglycerate kinase1.

Upon phagocytosis, *M. tuberculosis* is delivered to the phagosome where it resides in an infected macrophage. The phagosome is a nutrient deficient vacuole where mycobacteria are denied access to the more commonly used carbon sources. To facilitate adaptation, *M. tuberculosis* readily realigns its metabolism once inside the host with an increased preference for lipids as carbon substrates^1^. Sequencing of the *M. tuberculosis* genome has revealed the potential of *M. tuberculosis* to do so, with the identification of at least 250 genes potentially involved in lipid metabolism, a number that is unrivalled so far in the prokaryotic world^2^. The pathogen also induces lipid body (LB) accumulation in the macrophage which then differentiate into Foamy macrophages (FMs), ensuring an abundant supply of the lipid substrates^3^,^4^. The lipid loaded FMs ensure a privileged existence for the bacilli not only through a ready provision of nutrients but also, by conferring protection against bactericidal activities like respiratory burst, autophagy and lysosomal acidification^4^,^5^. The ability to cause FM differentiation is exclusive to virulent strains of *M. tuberculosis*^4^,^5^. The FMs lie at the heart of the disease and are responsible for the first appearance of the disease as lipid pneumonia^6^. The death of the FMs found at the centre of granulomas causes lipid accumulation as caseous debris which induces cavitation and release of bacteria for further dissemination^3^.

The *M. tuberculosis* virulence factor, early-secreted antigenic target (ESAT-6) mediates mycobacterial virulence by playing important roles in cellular invasion, escape from phagolysosomes, cell-to-cell spread and dissemination of *M. tuberculosis* and immunomodulation^7^,^8^,^9^,^10^. ESAT-6 along with its entire secretory apparatus are encoded by a region in the *M. tuberculosis* genome known as the region of difference 1 (RD1) and this region is conserved in several mycobacterial species including *M. tuberculosis*, *M. marinum* and *M. bovis*^11^. Recently ESAT-6 has also been identified as the key mycobacterial effector responsible for FM differentiation^4^. The FM differentiation pathway involves the activation of the anti- lipolytic G- protein coupled receptor, GPR109A in macrophages infected with virulent strains of *M. tuberculosis*, which prevents triglyceride turnover and thus, allowing lipid accumulation to occur in the form of lipid bodies (LBs) which are responsible for the foamy appearance. Activation of GPR109A occurs in response to the increased synthesis and secretion of its natural ligand, the ketone body 3- hydroxy butyrate (3- HB), a process largely mediated by ESAT-6 in *M. tuberculosis* infected macrophages. Although the conventional pathway for ketone body synthesis is through fatty acid oxidation^12^, 3-HB is synthesized through a non-classical pathway in *M. tuberculosis* infected macrophages where the glycolytic pathway is involved^4^. Also, glucose uptake by macrophages is enhanced in the presence of ESAT-6^4^, a phenomenon which is also seen upon infection of macrophages with virulent strains of *M. tuberculosis*^13^. It is believed that the increased glucose uptake coupled to metabolic flux perturbations causes differentiation of macrophages into FMs^4^.

In the present study, we sought to elucidate the metabolic perturbations induced in the macrophage by ESAT-6 which account for the differentiation to the foamy phenotype. We identified the glucose transporter (GLUT) employed for the ESAT-6 mediated increase in glucose uptake and made mass spectrometry (MS) - based concentration measurements of the glycolytic pathway intermediates in the presence of ESAT-6. The steady state metabolite concentrations were used in a mathematical model of glycolysis to estimate the rates of reaction for the various steps in the glycolytic pathway. We were also able to validate experimentally the rate changes for two separate steps in the glycolytic pathway, as suggested by the mathematical model. Subsequently in a co- immunoprecipitation experiment we picked up interactions between ESAT-6 and two glycolytic enzymes. The present study improves significantly, our knowledge of the *M. tuberculosis* induced FM differentiation process.

## Results

### Enhanced glucose uptake in the presence of ESAT-6 is GLUT-1 dependent

**Figure 1:**
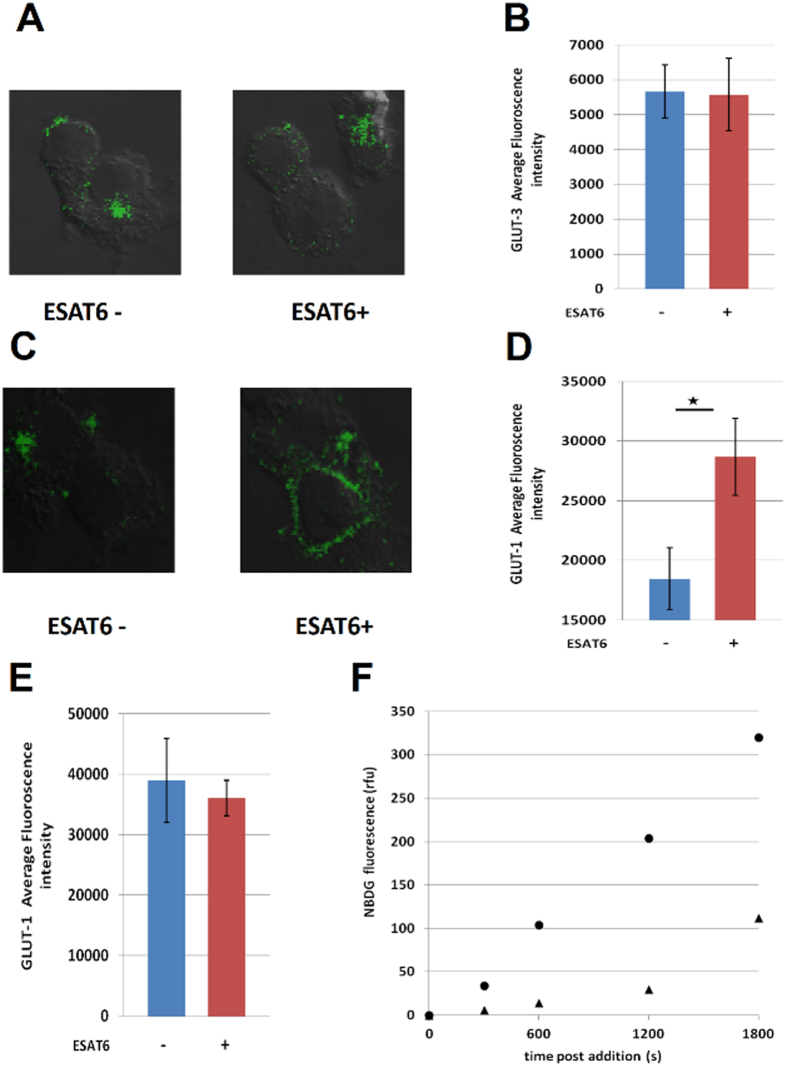
GLUT-1 dependent glucose uptake. (**A–D**) PMA- differentiated THP-1 cells were either left untreated (ESAT6−) or treated with 5 μg/ml rESAT6 (ESAT6+) for 3 h. Cells were then fixed and stained with anti- GLUT3/anti- GLUT1 antibody followed by FITC- labeled secondary antibody and examined under the laser-scanning confocal microscope at X 60 magnification. The images are represented as an overlay of the image obtained in the green channel (excitation 488 nm) over the Differential Interference Contrast (DIC) image of the same field. The bar plots are a quantitative representation of the average fluorescence intensity/cell values (±S.E) obtained from at least 50 cells from two separate experiments (significance, *P ≤ 0.01, unpaired t-test). (**A,B**) Cells stained for cell surface GLUT-3 and no effect is seen upon rESAT-6 addition. (**C,D**) Cells stained for cell surface GLUT1 and the cell surface expression is enhanced in cells treated with rESAT-6. (**E**) Cells were gently permeabilized with Triton X- 100 (0.2% v/v) and stained with anti- GLUT1 antibody followed by FITC- labeled secondary antibody to label total cellular GLUT1. The average fluorescence intensity/cell was quantified from the acquired images and the bar plot shows that treatment with rESAT-6 did not affect the cellular GLUT1 levels. Values are the mean (±) S.E. obtained from at least 50 cells from two separate experiments. (**F**) THP-1 cells were either left untreated (▲) or treated with rESAT-6 for 3 h (•) and incubated with 2-NBDG for 0, 5, 10, 20 and 30 minutes. The fluorescence of the internalized 2-NBDG was measured at 535 nm. The rate of NBDG uptake for untreated cells was, A = 0.058 and for cells treated with rESAT-6, A = 0.18 as calculated from the graph.

The facilitative glucose transporters GLUT1, GLUT3 and GLUT5 are expressed in the cell membranes of THP-1 macrophages which mediate glucose uptake^14^. We treated PMA- differentiated THP-1 cells with rESAT6 and measured the cell surface expression of GLUT1 and GLUT3 through a laser- scanning confocal microscope. Although the cell surface expression levels of GLUT3 did not change upon rESAT-6 treatment (Fig. 1A,B), there was a significant enhancement in the cell surface expression of the glucose transporter GLUT-1 upon rESAT-6 treatment (Fig. 1C,D). Also, this process was specific to ESAT-6 as the cell surface expression of GLUT-1 did not change in the presence of CFP-10, a mycobacterial secretory protein known to bind ESAT-6 (see Supplementary Fig. S1 online). Importantly the total amount of GLUT-1 protein did not increase upon rESAT-6 stimulation (Fig. 1E, see supplementary Fig. S1 online) and it was only the translocation of the protein from the cytosol to the cell membrane that was enhanced by rESAT-6 to increase glucose uptake by THP-1 macrophages. We next monitored the differential rates of glucose uptake between untreated THP-1 macrophages and rESAT-6 treated THP-1 macrophages by measuring the rate of uptake of 2-NBDG - a fluorescent analog of glucose. The 2- NBDG uptake rate for rESAT-6 treated THP-1 macrophages was nearly 3 folds higher than the basal uptake rate of THP-1 macrophages (Fig. 1F).

### Macrophage glycolysis is perturbed by ESAT-6

To determine the downstream consequences of the enhanced glucose uptake mediated by ESAT-6, we took THP-1 cells and incubated them overnight in glucose free RPMI supplemented with ^13^C_6_-glucose. This was done to ensure that the metabolites subsequently measured will all be carrying the ^13^C_6_- label that will originate from glucose only. The cells were then left untreated or treated with rESAT-6, following which metabolites were extracted and their concentrations quantified using MS. The addition of rESAT-6 to THP-1 macrophages had a profound effect on the glycolytic intermediates Glucose-6- phosphate (G6P), Dihydroxyacetone phosphate (DHAP), Phosphoglycerate (2PG/3PG) and Phosphoenol pyruvate (PEP), the concentrations being approximately 3 folds- higher (7 folds- for DHAP) in the presence of rESAT-6 (Table 1). Although Pyruvate concentration remained the same, the levels of Acetyl CoA (AcCoA) were again 3 folds- higher in THP-1 cells that were treated with rESAT6. The concentration of Oxaloacetate (OAA) was similar between cells that were left untreated (2.25 ± 0.3 μM) or treated with rESAT-6 (2.1 ± 0.1 μM). This is in concordance with our earlier findings^4^ that AcCoA concentration increases in the presence of ESAT-6 (also in macrophages infected with virulent strains of *M. tuberculosis*) thus favoring a shift towards a higher AcCoA/OAA ratio, a key signal for the synthesis of the ketone body, 3-hydroxy butyrate (3-HB)^15^. The citrate concentration did not change in the presence of rESAT-6 (Table 1). Although, ESAT-6 is known to be a pore- forming protein^7^, cells were mostly viable at the concentration of rESAT-6 used (see Supplementary Fig. S2 online).

### Mathematical model for glycolysis and rate predictions

**Figure 2:**
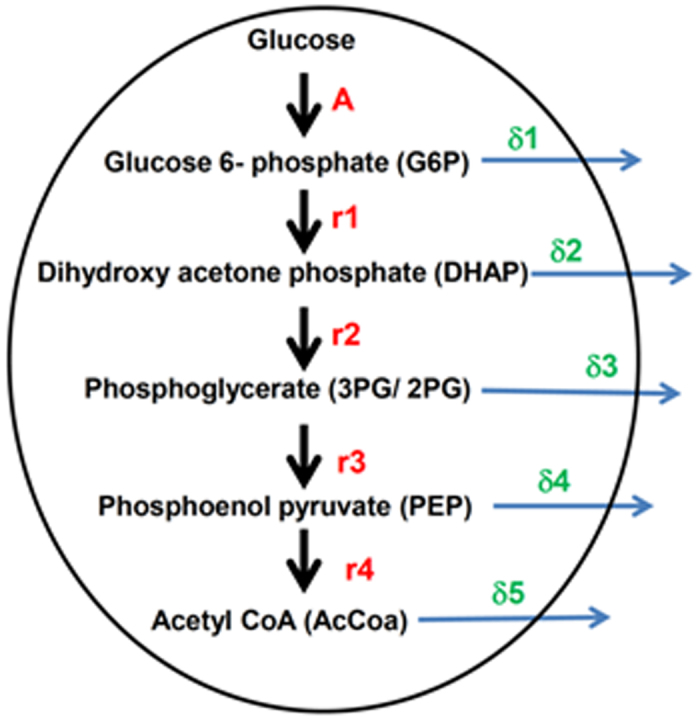
Mathematical model for glycolysis. Glucose is taken up and converted to G6P at a rate (**A**) which is then converted to DHAP at a rate ***r***_***1***_. Further downstream DHAP is converted to 3PG/2PG at a rate ***r***_***2***_, which in turn is converted to PEP at a rate ***r***_***3***_. Finally AcCoA is formed at a rate ***r***_***4***_. At each step, there is an out flux of the metabolites to other metabolic pathways, the rates of which are denoted by **δ**_***i***_, *i* = 1, 2, 3, 4, 5.

We constructed a mathematical model for the glycolytic pathway using the five most perturbed metabolites viz Glucose-6-phosphate (G6P), Dihydroxy acetone phosphate (DHAP), Phosphoglycerate (3PG/2PG), Phosphoenol pyruvate (PEP) and AcetylCoA (AcCoA) as the five variables for the model. We assumed that glucose is taken up and converted to G6P at a rate ***A***, which is then converted to DHAP at a rate ***r***_***1***_. Further downstream DHAP is converted to 3PG/2PG at a rate ***r***_***2***_, which in turn is converted to PEP at a rate ***r***_***3***_. Finally AcCoA is formed at a rate ***r***_***4***_. Here we also assumed that at each step, there is an out flux of the metabolites to other metabolic pathways, the rates of which are denoted by **δ**_***i***_, *i* = 1, 2, 3, 4, 5. The mathematical model is discussed in greater detail in the Supplementary information section. The model is represented in Fig. 2.

However, there was a limitation here i.e., the number of equations (considering each steady state expression as one equation) was less than the number of parameters that needed to be determined. So we had to feed in some arbitrary value for a few parameters which were **δ**_**1**_ = 0.001, **δ**_**2**_ = 0.0002, **δ**_**4**_ = 0.0. Also, the values for **A** were experimentally determined by measuring the glucose uptake rates (Fig. 1F), and were 0.058 and 0.18 for THP-1 cells left untreated (−rESAT-6) or treated with rESAT-6 (+rESAT-6) respectively. The remaining parameters were determined after solving the steady state expressions and are outlined in Table 2. The addition of rESAT-6 to THP-1 macrophages causes differential modulation of the glycolytic pathway where ***r***_***3***_*, **r***_***4***_ and **δ**_**5**_ are up-regulated in the presence of rESAT-6 while the rates ***r***_***2***_ and **δ**_**3**_ are attenuated (Table 2).

### Enolase1 and Pyruvate dehydrogenase enzyme activities are enhanced by ESAT-6

**Figure 3:**
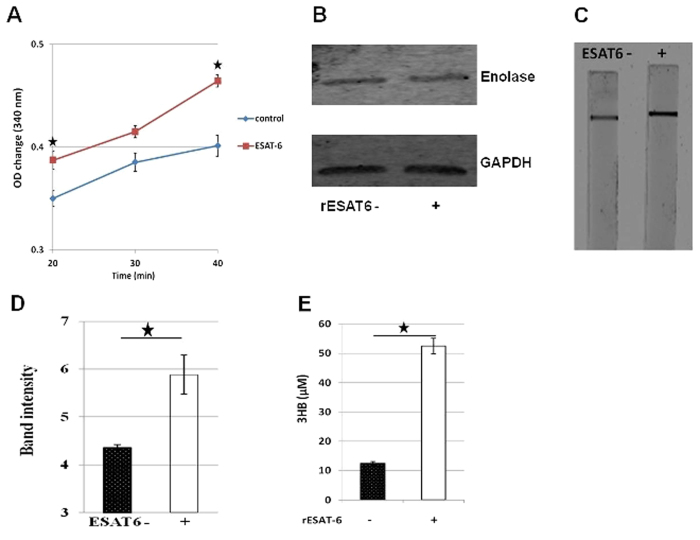
ESAT-6 enhances Enolase1 and Pyruvate dehydrogenase enzyme activities. (**A**) PMA- differentiated THP-1 cells were either left untreated (control) or treated with rESAT6 (ESAT-6) for 3 h. Enolase 1 activity was measured using the ENO1 Human Activity Assay Kit (Abcam) by monitoring for NADH consumption in a coupled reaction as decrease in absorbance at 340 nm. Enolase 1 activity was higher in cells treated with r-ESAT-6. n = 3, mean ± S.D.; significance, *P ≤ 0.05 (unpaired t-test). (**B**) Western blot analysis for measuring intracellular Enolase-1 protein levels in THP-1 macrophages in the absence (rESAT-6-) and in the presence of rESAT-6 (rESAT-6+). GAPDH was used as a loading control. (**C**) PMA- differentiated THP-1 cells were either left untreated (ESAT6−) or treated with rESAT6 (ESAT6+) for 3 h. Pyruvate dehydrogenase (PDH) activity was measured using the Pyruvate dehydrogenase (PDH) Enzyme Activity Dipstick Assay Kit (Abcam) where NADH produced by PDH activity reaction is coupled to the reduction and precipitation of a colored dye on the dipstick. Greater amount of precipitate formed when THP-1 macrophages had been treated with rESAT-6 indicating heightened PDH activity. (**D**) The dipstick was scanned using the Odyssey image scanner (Licor) and the images were analyzed using the Odyssey version 3.0 software to calculate the intensity of the precipitate band. n = 3, mean ± S.D., significance, *P ≤ 0.05 (unpaired t-test). (**E**) Effect of exogenous addition of rESAT-6 protein (rESAT-6+) on 3HB production by THP-1 cells measured at 48 h. n = 3, mean ± S.D.; significance, *P ≤ 0.05 (unpaired t-test).

The rate predictions obtained from the mathematical model seemed to suggest an increase in the rate of conversion of 2PG/3PG to PEP (***r***_***3***_), conversion of PEP to AcCoA (***r***_***4***_) and out flux of AcCoA (**δ**_**5**_) in rESAT-6 treated cells. Enolase1 (ENO1) is the enzyme responsible for the catalysis of the conversion of 2-phosphoglycerate (2-PG) to phosphoenolpyruvate (PEP) and we measured the activity of the native enzyme in cell extracts of THP-1 macrophages that were either left untreated (control) or treated with rESAT-6 (ESAT-6). The ENO-1 activity was found to be significantly higher in the presence of rESAT-6 (Fig. 3A) and this validated the model predicted increase in ***r***_***3***_ in the presence of ESAT-6. Importantly, Enolase1 protein levels were found to be similar between control and rESAT-6 treated cells (Fig. 3B). The conversion of PEP to AcCOA (***r***_***4***_) is a two- step process where Pyruvate kinase first catalyzes the conversion of PEP to Pyruvate which subsequently becomes the substrate of the Pyruvate dehydrogenase (PDH) multi-enzyme complex for conversion into AcCoA. Since the Pyruvate concentration does not change upon ESAT-6 treatment (Table 1), we reasoned that the predicted increase in ***r***_***4***_ could be because of heightened PDH activity. To test this we measured the PDH activity in cell extracts of THP-1 macrophages in the absence (ESAT6−) or presence of rESAT-6 (ESAT6+) using a dip- stick assay. As expected PDH activity was found to be higher in cells that had been treated with rESAT-6 (Fig. 3C,D) resulting in increased conversion of Pyruvate to AcCoA. The increased out flux of AcCoA (**δ**_**5**_) in rESAT-6 treated cells in the absence of any significant increase in citrate concentration (Table 1), could be explained by the increased synthesis of the ketone body 3-HB (Fig. 3E), which has also been shown previously^4^.

### ESAT-6 induced buildup of DHAP leads to increased Triglyceride (TAG) levels

**Figure 4:**
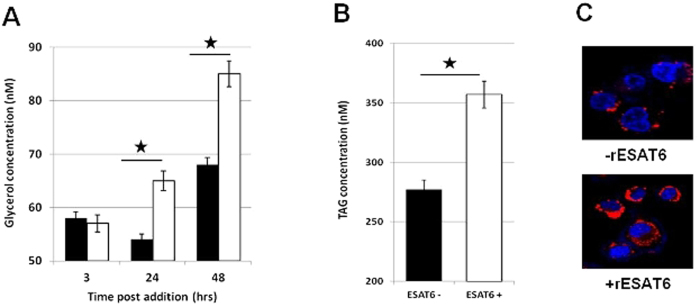
ESAT-6 increases intracellular Triglyceride. (**A**) PMA- differentiated THP-1 cells were either left untreated (black bars) or treated with rESAT6 (white bars) for 3, 24 and 48 hrs and intracellular glycerol concentration was estimated. The glycerol concentration was significantly higher in cells that had been treated with rESAT-6 for 24 and 48 hrs. n = 3, mean ± S.D.; significance, *P ≤ 0.01 (unpaired t-test). (**B**) PMA- differentiated THP-1 cells were either left untreated (black bar) or treated with rESAT6 (white bar) for 48 hrs and intracellular Triglyceride (TAG) concentration was estimated. rESAT-6 treated cells had a higher TAG concentration compared to untreated cells. n = 3, mean ± S.D.; significance, *P ≤ 0.01 (unpaired t- test). (**C**) PMA-differentiated THP-1 cells were left untreated (−rESAT6), or treated with rESAT6 (+rESAT6) for 48 hrs and then stained with Lipid Tox (red) at 48 h to score for lipid body formation. Cell nuclei were counterstained with DAPI (blue). rESAT-6 induces lipid body formation in macrophages and causes foamy macrophage differentiation. The fluoroscence intensity of lipid bodies averaged over 50 cells is 2695 ± 320 for untreated cells and 5688 ± 487 for ESAT-6 treated cells.

The rate of conversion of DHAP to Phosphoglycerate (***r***_***2***_) is attenuated in the presence of rESAT6 (Table 2). This observation was surprising, given the overall increased flux through the glycolytic pathway induced by ESAT-6. However, DHAP is an important metabolite in the TAG synthesis pathway. The enzyme glycerol-3-phosphate dehydrogenase (GPDH) catalyzes the reversible conversion of DHAP to glycerol 3-phosphate and thus links carbohydrate metabolism to lipid metabolism^16^. Also, virulent strains of *M. tuberculosis* have been shown to induce *de novo* lipid biosynthesis in host cells^13^. Therefore, to test if the attenuation of ***r***_***2***_ in the presence of ESAT-6 had any effects on lipid metabolism, we measured intracellular glycerol concentrations at various time intervals post addition of rESAT-6. The glycerol concentration was significantly higher in cells that had been treated with rESAT-6 (Fig. 4A). Next, to confirm that the increased glycerol levels reflected at the level of TAG, we measured the intracellular TAG concentration in the presence or absence of rESAT-6 and the increased glycerol levels did indeed translate into higher TAG levels in the presence of rESAT-6 (Fig. 4B) and finally into LBs (Fig. 4C) which are a hallmark of FMs.

### ESAT-6 interacts with the glycolytic enzymes Enolase1 and Phosphoglycerate kinase 1

**Figure 5:**
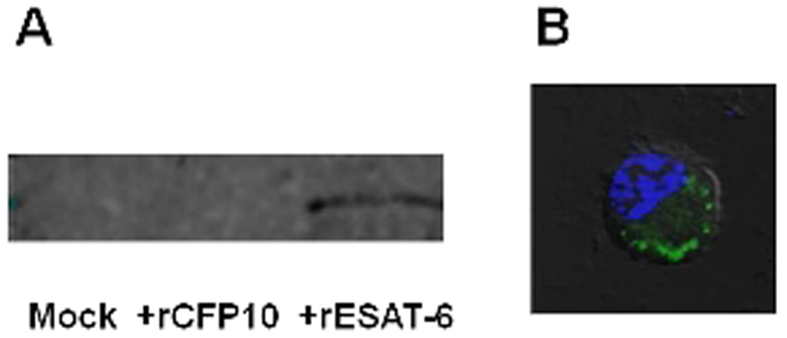
ESAT-6 specific interactions. (**A**) THP-1 macrophages were left untreated (mock) or incubated either with 5 μg/ml CFP-10 (+rCFP10) or 5 μg/ml ESAT-6 (+rESAT-6) for 1 hour. The cells were lysed and incubated with Anti- His_6_− tag mAb- Magnetic agarose beads to co-immunoprecipitate the interacting proteins with the His_6_− tagged bait protein and the samples were subjected to Western blot analysis for presence of Enolase-1. A protein band corresponding to Enolase1 was observed only when the co-immunoprecipitation was performed with ESAT-6, but not with CFP-10 or when no protein was present as bait, indicating specificity of the interaction. (**B**) PMA- differentiated THP-1 cells were treated with rESAT6 for 1 h. Cells were then fixed, gently permeabilized and stained with anti- ESAT-6 antibody followed by FITC- labeled secondary antibody and examined under the laser-scanning confocal microscope at X 60 magnification. Cell nuclei were counterstained with DAPI (blue). The represented image is an overlay of the image obtained in the green channel (excitation 488 nm) and in the blue channel (excitation 405 nm) over the Differential Interference Contrast (DIC) image of the same field. Intracellular ESAT-6 can be clearly seen in the image.

Since ESAT-6 is a mycobacterial secretory protein released into the cytosol of infected macrophages^4^, it seemed likely that some of the metabolic flux perturbations caused by ESAT-6 were due to interactions between ESAT-6 and one or more of the macrophage proteins. To decipher the host interactome of ESAT-6, THP-1 macrophages were incubated with rESAT-6 and after a gentle lysis of the cells, the His_6_− tagged rESAT-6 and any interacting protein(s) were co –immunoprecipitated using Anti- His- tag mAb- Magnetic agarose beads. The co- immunoprecipitated proteins were subjected to proteolytic digestion followed by 2D LC-MS/MS mass spectrometric analysis. The eight macrophage proteins that co –immunoprecipitated with rESAT-6 are listed in Table 3. Two of these proteins are the glycolytic enzymes, Enolase1 and Phosphoglycerate kinase 1 (PGK1). To validate our findings we performed western blot analysis of the mock (−rESAT-6) and ESAT-6 (+rESAT-6) Co-IP fractions and probed for Enolase1. In addition, to ensure further specificity of our findings we included a Co-IP fraction that was performed using another secretory protein of M. tuberculosis, CFP-10 (+rCFP-10). The interaction between ESAT-6 and Enolase1 was indeed specific as Enolase-1 was detected only in the ESAT6 Co-IP fraction but not in the mock or CFP-10 Co-IP fractions (Fig. 5A). Since, we were using extracellular ESAT-6 protein in our study, to be sure that the protein was entering the macrophages and was indeed cytosolic, we performed antibody staining (anti-ESAT6) in cells that had been treated with rESAT-6 and analyzed the cells by confocal microscopy. We could detect intracellular ESAT-6 (Fig. 5B) and further validation was provided by MS- based detection of ESAT-6 in the rESAT-6 Co-IP fraction (not shown).

## Discussion

Host-pathogen interactions are critical for determining the clinical outcome of an infection process. The pathogen employs multiple strategies to shift the balance in its favor. One such strategy employed by the TB pathogen, *M. tuberculosis* is the infection induced differentiation of the macrophage into lipid loaded FMs. The FMs form a safe haven for the intracellular mycobacteria where on one hand it has easy access to nutrients in the form of lipids/fatty acids and on the other, it is protected against antimicrobial activities of the macrophage like respiratory burst, autophagy and lysosomal acidification^4^,^5^. The *M. tuberculosis* virulence factor ESAT-6, is the mycobacterial molecular determinant that mediates the FM differentiation process^4^. This activity is in addition to the other well appreciated roles of ESAT-6 in promoting *M. tuberculosis* virulence^7^,^8^,^9^,^10^. Preliminary analysis revealed that ESAT-6 induces increased glucose uptake and production of 3-HB (the natural ligand for the anti-lipolytic receptor GPR109A) by macrophages, to cause FM differentiation^4^. However, the intermediary steps involved in this process were largely unknown.

Here, we show that the ESAT-6 dependent enhancement of glucose uptake by macrophages is largely mediated by the increased surface expression of the glucose transporter, GLUT1. Importantly, this happens due to increased translocation of GLUT1 from the cell cytosol to the membrane, with the total amount of GLUT1 remaining unchanged in cells upon ESAT-6 treatment. The rate of glucose uptake in the presence of ESAT-6 was found to be 3 folds- higher compared to the basal rate of glucose uptake by differentiated macrophages. The downstream effects of the enhanced glucose uptake involved increase in the intracellular concentrations of the glycolytic intermediates Glucose-6- phosphate (G6P), Phosphoglycerate (2PG/3PG) and Phosphoenol pyruvate (PEP) which were approximately 3 folds- higher compared to basal levels. DHAP concentration was even higher (7 folds-) in the presence of ESAT-6. Although no effects were seen on Pyruvate concentration, the levels of Acetyl CoA (AcCoA) were increased by a factor of three. Increase in the AcCoA/OAA ratio is the inflection point for the synthesis of the ketone body, 3-hydroxy butyrate (3-HB)^15^. The increase in AcCoA concentration in the presence of ESAT-6 without any significant increase in OAA concentration, thus favored the AcCoA/OAA ratio in the direction of 3-HB synthesis. Also pertinent here is the observation that the citrate concentration did not change in the presence of ESAT-6, thus allowing for the increased AcCoA to be shunted entirely for 3-HB synthesis.

Using a mathematical model of glycolysis we further found that the rates of conversion of 2PG/3PG to PEP, conversion of PEP to AcCoA and out flux of AcCoA were enhanced in the presence of ESAT-6. While the increased out flux of AcCoA could be explained by the increased synthesis of the ketone body 3-HB, the former observations were explained by the increased activities of the enzymes, Enolase 1 and Pyruvate dehydrogenase in the presence of ESAT-6. Importantly the increased rate of conversion of 2PG/3PG to PEP in the presence of ESAT-6 was due to enhanced Enolase1 activity only and not due to increase in the protein amount. The rates of conversion of DHAP to Phosphoglycerate and the out flux of Phosphoglycerate were however, attenuated in the presence of ESAT6. The attenuation of turnover of DHAP although surprising at first glance, seemed to suggest a link with lipid metabolism as DHAP is an important metabolite in the TAG synthesis pathway. The buildup of DHAP concentration due to slower conversion to Phosphoglycerate contributed to increased intracellular glycerol and TAG concentrations. Virulent strains of *M. tuberculosis* are known to induce *de novo* lipid biosynthesis in host cells^13^ and a major part could be ESAT-6 mediated.

Mathematical model simulations provided further validation for the fact that the observed effects were indeed due to active modulation of the glycolytic pathway by ESAT-6, and not simply because of increased substrate availability due to enhanced glucose uptake. Simulating increased glucose uptake by THP-1 macrophages (in the absence of ESAT-6) predicted increase in metabolite concentrations similar to that obtained with ESAT6, with the notable exception being DHAP where the predicted concentration was half of the actual concentration obtained in the presence of ESAT6 (Table 4a). Alternatively, DHAP buildup (to the extent obtained in presence of ESAT6) was simulated in THP-1 macrophages which predicted AcCoA concentration to be twice of the actual concentration obtained in the presence of ESAT-6 (Table 4b). This clearly suggested that the metabolic flux perturbations are not a linear effect of increased glucose availability, but involve active modulation of the glycolytic pathway by ESAT-6.

ESAT-6 is a 6 kDa secretory protein of *M. tuberculosis* that is actively secreted by the bacilli into the host cell cytoplasm following infection of macrophages with virulent strains of *M. tuberculosis*^4^. Some of it also finds it way into the extracellular medium^4^. Release into the host cytosol brings ESAT-6 into close proximity of several cytosolic proteins. Establishment of physical contacts between ESAT-6 and one or more of the cytosolic proteins will in turn induce structural and/or functional changes accounting for the observed phenotype. Previous studies have shown interactions between ESAT-6 and the host proteins TLR-2^10^, ADAM9 metalloprotease^17^ and syntenin-1^18^. Recently, *M. tuberculosis* ESAT-6 has also been shown to interact with Beta-2-Microglobulin (β2M) and this interaction negatively affects the antigen presentation function of the macrophage^19^. In the present study we detected unique interactions between ESAT-6 and eight macrophage proteins namely Enolase1, PGK1, Myosin heavy chain MYH9, 68 kDa TBP- associated factor, Alpha1 actinin, Profilin-1, Nucleolin and the Eukaryotic translation initiation factor 1A. The interactions between ESAT-6 and the glycolytic pathway enzymes, Enolase-1 and PGK1 could be a prerequisite for inducing the metabolic flux perturbations and might involve active modulation of the enzyme activities due to binding of ESAT-6. The interactions between ESAT-6 and the other host proteins might be contributing to mycobacterial virulence through, yet unknown mechanisms.

In conclusion, the present study provides a significant advancement in our understanding of host- pathogen interactions during *M. tuberculosis* infection especially in context of the Foamy macrophage (FM) differentiation process. A two- pronged strategy which includes simultaneous buildup of DHAP (for Triglyceride synthesis) and AcCoA (for synthesis of 3-HB, ligand for the anti-lipolytic GPR109A) mediated by ESAT-6, forms the molecular mechanism driving this process, critical for *M. tuberculosis* pathogenesis. We believe such understanding would help us to design novel small molecule inhibitors for tuberculosis therapy.

## Methods

### Cells, Cultures, and Media

The human promonocytic cell line THP1 (American Type Culture Collection) was used in this study. THP1 cells were cultured in RPMI 1640 (Invitrogen) supplemented with 10% FCS (Hyclone) and were maintained between 2 and 10 × 10^5^ cells/ml at 37 °C in a humidified, 5% CO_2_ atmosphere. Cells were seeded in 6-well plates at 2 × 10^6^ cells/well and treated with 30 ng/ml phorbol 12-myristate 13-acetate (PMA; Sigma) overnight; cells were allowed to adhere and become differentiated into macrophages at 37 °C in a humidified, 5% CO_2_ incubator.

### Expression and purification of recombinant ESAT-6 (rESAT-6)

ESAT-6 protein was expressed as a derivative with C-terminal hexa-histidine tag using pVNLMTBESAT64102 as described before^20^ except for the change in growth conditions and purification steps. The initial culture was grown in non-inducing MDAG medium and protein was expressed using super broth medium. During purification, Ni-Sepharose Fast Flow (NiFF) column was used in place of Ni-Sepharose High performance (NiHP) and 1X TLB (Tris Loading Buffer; 50 mM Tris, pH 7.5 containing 0.5 M NaCl) was used in place of 1X LB (Loading Buffer; 20 mM sodium phosphate, pH 7.6 containing 140 mM NaCl). The overall purification contained three-steps as described before^20^.

### Confocal microscopy

PMA-differentiated THP1 cells were seeded at a density of 0.3 × 10^6^ cells/coverslip, onto 12-mm diameter glass coverslips in a 24-well tissue culture plate. rESAT-6 (His_6_− ESAT-6; recombinant ESAT-6) was added to the differentiated THP1 cells at a final concentration of 5 μg/ml for 3 h^4^. Cells were washed and fixed with 3.7% paraformaldehyde (Sigma-Aldrich). Blocking was performed using 3% (w/v) BSA for 1 h. This was followed by primary anti GLUT-1/GLUT3 (Abcam) antibody staining for 1 h and secondary goat anti- mouse FITC (Abcam) for 1 h. The coverslips were washed thoroughly with PBS and were mounted onto glass slides with Slowfade reagent (Invitrogen). Four fields were acquired randomly from each set of stained cells with a Nikon EclipseTi-E laser-scanning confocal microscope equipped with 60X/1.4 numerical aperture oil planapochromat differential interference contrast objective lens using argon laser (excitation at 488 nm and emission at 515 nm). All images were acquired using the software EZ-C1 3.8 and analyses were performed using the software Image-Pro Plus version 6.0.

### Glucose uptake assay

THP-1 cells were either left untreated or treated with rESAT-6 (5 μg/ml) for 3 h. Cells were initially washed with glucose free RPMI followed by addition of glucose free RPMI supplemented with 200 μM 2-NBDG for 0, 10, 20 and 30 minutes. Subsequently, the labeling media was removed, cells were washed in glucose free RPMI and lysed in 200 μl of a non-interfering buffer (1% Nonidet P-40, 1% sodium deoxycholate, 40 mM KCl, 20 mM tris pH7.4). The lysate was centrifuged at 13000 g at 4 °C for 5 minutes to remove debris. Fluorescence of the internalized glucose was measured on a flourimeter at 535 nm (excitation wavelength 485 nm).

### Metabolite extraction and mass spectrometry based quantification

Labeled RPMI medium was prepared by adding 2 g/liter of ^13^C_6_-glucose to glucose- free RPMI and differentiated THP-1 cells (5 × 10^6^) were incubated in the labeled medium supplemented with 10% FCS for 16 h at 37 °C in a humidified, 5% CO_2_ atmosphere. The cells were then either left untreated or treated with rESAT-6 (5 μg/ml) for 3 h. Metabolic reactions were quenched by adding chilled (−75 °C) mixture of Methanol: water (80:20, v/v). After incubation at −75 °C for 10 minutes the cells were scrapped from the culture dish and collected. The cell suspension was then vortexed, followed by centrifugation at 6000 g for 5 minutes at 4 °C, and the supernatant was collected. The pellet was re-extracted two more times with 80% methanol at −75 °C. The three extractions were pooled, centrifuged at 13000 g for 5 minutes to remove any debris and dried under a Nitrogen stream. The dried samples were resuspended in MS-grade water, vortexed and centrifuged at 13000 g, 4 °C for 10 minutes. Supernatants were used for the LC-MS/MS analysis. High-pressure liquid chromatography (HPLC) was performed on an Agilent 1260 infinity Binary HPLC (Agilent technology) equipped with a degasser, and an auto sampler. In all, two types of columns were used in the study- i) Aminopropyl ii) C-18. The auto sampler was maintained at 4 °C to ensure sample stability. A flow rate of 200 μl/min and sample injection volume of 20 μl was maintained in the study. The HPLC was coupled with a hybrid 4000 QTRAP (AB SCIEX) with a Turbo V ESI ionization source interface, and a computer platform equipped with a Solution Analyst software version 1.5 (ABSciex) which was used for data acquisition and processing. Metabolites were quantified by employing concentration dependent standard curves for each metabolite.

### Enolase1 activity, Pyruvate dehydrogenase enzyme activity, and Glycerol and Triglyceride quantification

The assays were performed using ENO1 Human Activity Assay Kit (Abcam), Pyruvate dehydrogenase (PDH) Enzyme Activity Dipstick Assay Kit (Abcam), Free Glycerol Detection Kit (Abcam) and Triglyceride Quantification Kit (Abcam) respectively, as per the manufacturer’s instructions.

### Co- Immunoprecipitation (Co-IP) and protein identification by Mass spectrometry

PMA-differentiated THP1 cells (10 × 10^6^ cells) were incubated with rESAT-6 (5 μg/ml) for 1 h at 37 °C. The cells were washed extensively with chilled PBS to remove any extracellular rESAT-6. The cells were gently lysed with cold IP-lysis buffer (50 mM Tris.Cl, 150 mM NaCl, 1 mM EDTA, 1% Triton X-100, pH 7.4) and the cell lysate was centrifuged at 12000 g to separate the cell debris. The supernatant was incubated with Protein-A Dynabead for pre- clearing. The pre cleared cell lysate was incubated with Anti- His- tag mAb- Magnetic agarose beads (MBL) for 30 min at 4 °C with end over end mixing. The beads were collected by magnetic separation and washed repeatedly with cold IP-lysis buffer. The bead immobilized proteins were extracted in Acid Elution buffer (0.1 M Glycine.HCl, pH 3.0) and then neutralized with Alkaline Neutralization buffer (1 M Tris, pH 9.5). A mock Co-IP was done without the rESAT-6 to identify non-specific beads interactors. The Co-IP sample was lyophilized, and then re-suspended in 100 mM ammonium bicarbonate buffer. The proteins were then subjected to denaturation and reductive alkylation prior to digestion with trypsin.

Peptides were analyzed on a nano LC system (ABSciex) coupled to a triple TOF 5600 Mass Spectrometer (ABSciex). Each fraction was dissolved in 25 μl of 1A buffer (98% water, 2% ACN and 0.1% Formic acid) and two injections of 10 ul each was picked up by auto sampler and directly loaded on to nano LC trap column and then eluted out with the analytical chromolith column (particle size 5 μm, length 15 cm, ID 75 μm) which was directly mounted on the electro spray ion source. The total run time was 75 min with 52 min gradient from 5% to 90% acetonitrile in 0.1% formic acid at a flow rate of 500 nl/min. Ion spray voltage floating (IVSF) was set to 2300 & GS1 was set to 12. MS data was acquired in information-dependent acquisition mode using Analyst QS 1.6 software (ABSciex).

All automatic data analysis (MS and MS/MS) and database searching were conducted against the Uniprot database (version 04-25-2012) using the ProteinPilotTM software (version 4.0, revision 148085, Applied Biosystems) with the ParagonTM method. The raw peptide identification results from the ParagonTM Algorithm (Applied Biosystems) searches were further processed by the Pro GroupTM Algorithm (Applied Biosystems) within the Protein Pilot software before final display^21^. The parameters used for identification of the proteins includes: 1) Threshold of 5% accepted Global False discovery rate (G-FDR) proteins; 2) At least one peptide with 95% confidence for identification^21^. The false positive rates of the aforementioned filter criteria were all below 5%, estimated by using an individual reversed (decoy) sequence database. In brief, false positive rates were calculated by dividing the number of decoy hits by that of hits acquired in search against forward sequence database^22^.

## Additional Information

**How to cite this article**: Singh, V. *et al.* *M. tuberculosis* Secretory Protein ESAT-6 Induces Metabolic Flux Perturbations to Drive Foamy Macrophage Differentiation. *Sci. Rep.* **5**, 12906; doi: 10.1038/srep12906 (2015).

## Supplementary Material

Supplementary Information

## Figures and Tables

**Table 1:** Metabolite concentrations.

Metabolite	−rESAT-6	+rESAT-6
G6P	3.7 ± 0.3	14.5 ± 5.0
FBP	0.2 ± 0.02	0.3 ± 0.03
DHAP	24.7 ± 8.8	172.2 ± 23.3
3PG/2PG	164.2 ± 31.3	539.6 ± 70.4
PEP	56.7 ± 2.0	199.4 ± 19.5
Pyruvate	0.3 ± 0.1	0.2 ± 0.05
Citrate	35.7 ± 0.1	40.8 ± 0.3
AcCoA	1.2 ± 0.1	4.0 ± 0.2

PMA- differentiated THP-1 cells were either left untreated (−rESAT-6) or treated with rESAT6 for 3 h (+rESAT-6) and their metabolites were extracted and concentrations determined using Mass spectrometry. The concentrations are in μM for each metabolite. The values are mean ( ± ) S.D obtained from three separate experiments.

**Table 2:** Rate predictions.

	control	ESAT-6	ESAT-6/control
*r*_*1*_	0.014508	0.011385	0.8
*r*_*2*_	0.000903	0.00028	0.3
*r*_*3*_	0.0000354	0.0000895	2.5
*r*_*4*_	0.000104	0.000242	2.3
*δ*_*3*_	0.0001	0.00001	0.1
*δ*_5_	0.135116	0.268256	2.0

The steady state expressions were solved and the parameter values that were determined are listed in the table along with the ratios of the parameter value for rESAT-6 treated cells (ESAT-6) to the parameter values for untreated cells (control).

**Table 3:** ESAT-6 interactome.

No.	Accession #	Protein Name	Peptides (95%)
1	Q60FE2	MYH9 variant protein, Cellular myosin heavy chain, type A	4
2	K7EM90	Enolase 1	3
3	Q86X94	TATA box binding protein (TBP)-associated factor, 68 kDa	5
4	B7TY16	Actinin alpha 1 isoform 3	4
5	E7ERH5	Phosphoglycerate kinase 1	1
6	P07737	Profilin-1	2
7	P19338	Nucleolin	2
8	A6NJH9	Eukaryotic translation initiation factor 1A, Y-chromosomal	1

List of proteins that co- immunoprecipitated with rESAT-6.

**Table 4:** Mathematical simulations.

(a)			
Metabolite	−rESAT-6	+rESAT-6	Glucose uptake simulation
G6P	3.74	14.534	11.61
DHAP	24.6	172.24	76.34
2PG/3PG	164	539.58	508.97
PEP	56	199.41	173.79
AcCoA	0.43	1.80	1.33
			
**(b)**			
**Metabolite**	**−rESAT-6**	**+rESAT-6**	**DHAP buildup simulation**
DHAP	24.6	172.24	172.00
2PG/3PG	164	539.58	1146.67
PEP	56	199.41	391.54
AcCoA	0.43	1.80	3.01

(a) Glucose uptake by THP-1 macrophages (in the absence of rESAT-6) was simulated by giving A = 0.18 and keeping other parameters (*r*_*1–4*_ and ***δ***_*1–5*_) unchanged. (b) DHAP buildup in THP-1 macrophages (in the absence of rESAT-6) was simulated by fixing DHAP concentration at 172 μM and keeping other parameters (***r***_***1–4***_ and ***δ***_***1–5***_) unchanged.
