# Supplementary material for: M. tuberculosis Secretory Protein ESAT-6 Induces Metabolic Flux Perturbations to Drive Foamy Macrophage Differentiation
Source: Sci Rep. 2015 Aug 7;5:12906. doi: 10.1038/srep12906 (PMC5388048; doi:10.1038/srep12906)
Supplement: Supplementary Information [file srep12906-s1.pdf]

# SUPPLEMENTARY INFORMATION

# *M. tuberculosis* Secretory Protein ESAT-6 Induces Metabolic Flux Perturbations to Drive Foamy Macrophage Differentiation

Varshneya Singh[1], Charanpreet Kaur[2], Vijay K Chaudhary[2], Kanury VS Rao[3*] & Samrat Chatterjee[1*]

[1] Drug Discovery Research Centre, Translational Health Science & Technology Institute, Gurgaon-122016, India, [2] Department of Biochemistry, University of Delhi South Campus, New Delhi-110021, India, [3] Immunology Group, International Centre for Genetic Engineering and Biotechnology, New Delhi- 110067, India.

## SUPPLEMENTARY FIGURE S1

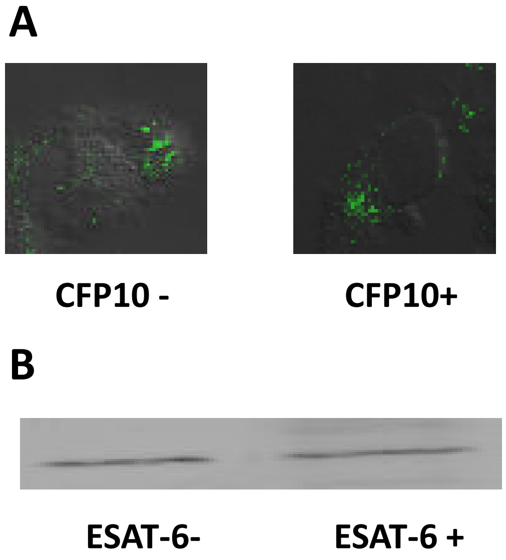


**SUPPLEMENTARY FIGURE S1. ESAT-6 specifically increases GLUT-1 membrane expression.** ***A,*** PMA- differentiated THP-1 cells were either left untreated (CFP10-) or treated with 5 µg/ml rCFP-10 (CFP10+) for 3h. Cells were then fixed and stained with anti- GLUT1 antibody followed by FITC- labeled secondary antibody and examined under the laser-scanning confocal microscope at X 60 magnification. The images are represented as an overlay of the image obtained in the green channel (excitation 488 nm) over the Differential Interference Contrast (DIC) image of the same field. No change in GLUT-1 cell surface expression is observed upon CFP-10 treatment. Average fluorescent intensity/cell: CFP10-: 17345$\pm$ 1148; CFP10+: 17556 $\pm$ 629. ***B,*** THP-1 macrophages were left untreated (ESAT-6-) or treated with ESAT-6 (ESAT-6+) for 3h following which cell lysates were generated and analyzed by western blot for GLUT1 levels. The total cellular GLUT1 levels were unchanged upon ESAT-6 treatment. The band intensity was 4.24 (ESAT-6-) and 4.16 (ESAT-6+).

# SUPPLEMENTARY FIGURE S2

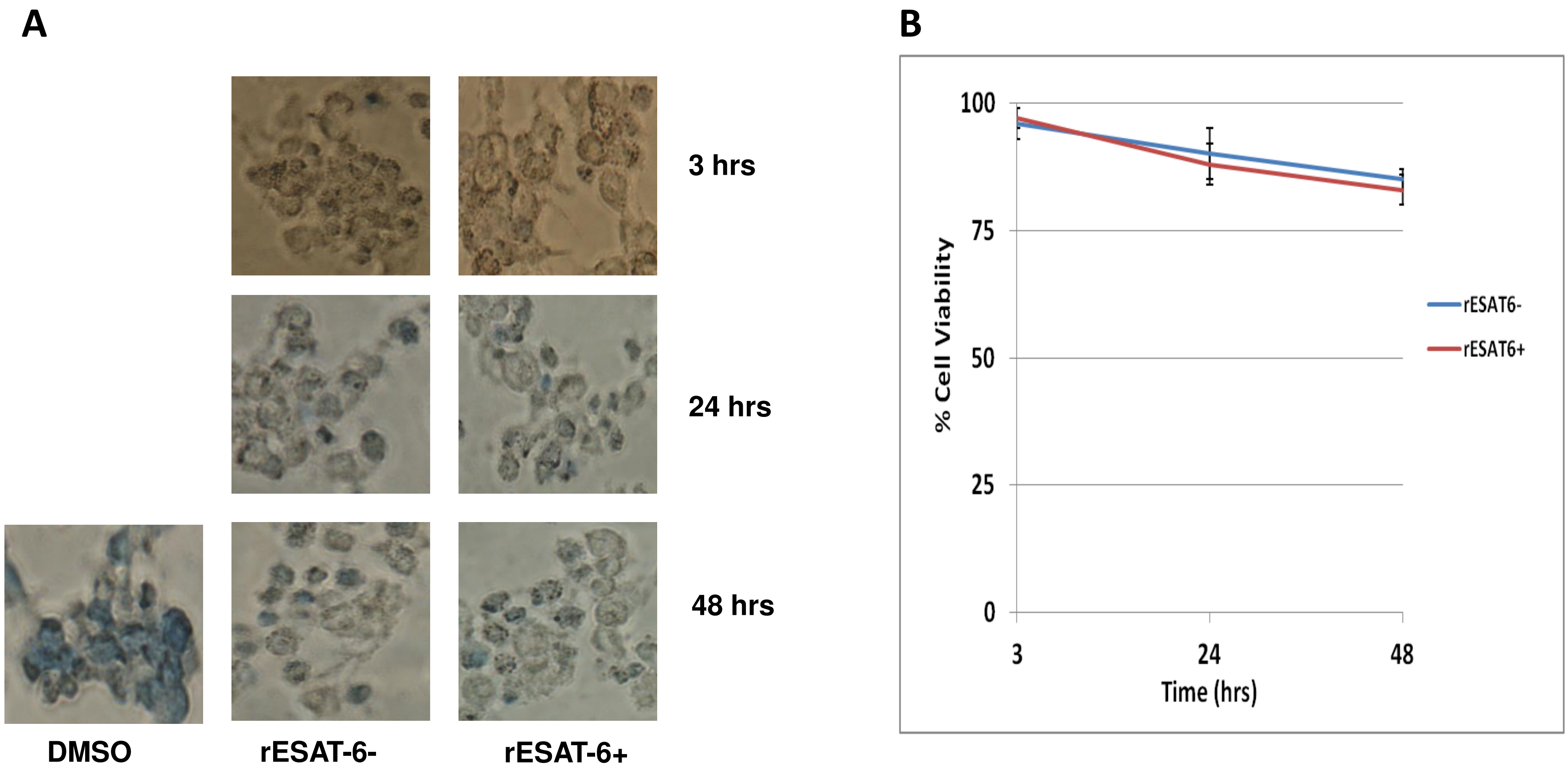


**SUPPLEMENTARY FIGURE S2. Effect of ESAT-6 on THP-1 cell viability.** ***A,*** PMA-differentiated THP-1 cells were either left untreated (rESAT-6-) or treated with 5 µg/ml rESAT-6(rESAT-6+) for 3h, 24h and 48h. Cells were stained with the vital dye, Trypan Blue to identify dead cells. DMSO- treated cells served as a positive control. ***B,*** MTT assay was performed to assess cell viability. Treatment of THP-1 macrophages with 5 µg/ml rESAT-6 has no significant effects on cell viability.

**Mathematical model of Glycolysis**

With the assumptions outlined in the text, we arrived at the following set of equations:

$$\frac{d[G6P]}{dt} = A - r_1[G6P] - \delta_1[G6P] \qquad (1)$$

$$\frac{d[DHAP]}{dt} = \frac{r_1}{4}2[G6P] - r_2[DHAP] - \delta_2[DHAP] \qquad (2)$$

$$\frac{d[PG]}{dt} = r_2[DHAP] - r_3[PG] - \delta_3[PG] \qquad (3)$$

$$\frac{d[PEP]}{dt} = r_3[PG] - r_4[PEP] - \delta_4[PEP] \qquad (4)$$

$$\frac{d[AcCoA]}{dt} = 10r_4[PEP] - \delta_5[AcCoA] \qquad (5)$$

where [G6P], [DHAP], [PG], [PEP] and [AcCoA] denote the concentrations of Glucose-6-phosphate (G6P), Dihydroxy acetone phosphate (DHAP), Phosphoglycerate (3PG/2PG), Phosphoenol pyruvate (PEP) and AcetylCoA (AcCoA) respectively.

The system has a single feasible interior steady state $E^* \equiv (G6P^*, DHAP^*, PG^*, PEP^*, AcCoA^*)$ with,

$$G6P^* = \frac{A}{r_1+\delta_1} \qquad (6)$$

$$DHAP^* = \frac{A-\delta_1 G6P^*}{r_2+\delta_2} \qquad (7)$$

$$PG^* = \frac{r_2 DHAP^*}{r_3+\delta_3} \qquad (8)$$

$$PEP^* = \frac{r_3 PG^*}{r_4+\delta_4} \qquad (9)$$

$$AcCoA^* = \frac{10 r_4 PEP^*}{\delta_5} \qquad (10)$$

The Jacobian matrix of the system around any arbitrary point (*G6P, DHAP, PG, PEP,* and *AcCoA*) is given by,

$$J = \begin{pmatrix} -(r_1+\delta_1) & 0 & 0 & 0 & 0 \\ \frac{r_1}{2} & -(r_2+\delta_2) & 0 & 0 & 0 \\ 0 & r_2 & -(r_3+\delta_3) & 0 & 0 \\ 0 & 0 & r_3 & -(r_4+\delta_4) & 0 \\ 0 & 0 & 0 & 10r_4 & -\delta_5 \end{pmatrix} \qquad (11)$$

The eigenvalues corresponding to the Jacobian matrix $J$, are - $(r_1+\delta_1)$, - $(r_2+\delta_2)$, - $(r_3+\delta_3)$, - $(r_4+\delta_4)$ and $-\delta_5$. Hence the system is always stable around the positive steady state. Thus the steady state analysis with its stability property gives a guarantee for the existence of a unique stable steady state which can be used for estimating the parameter values. So if the steady state value (i.e. the steady state metabolite concentrations) is known, the obtained steady state expression can be used for calculating the corresponding parameter values (i.e. rates of reactions).
